# Deep brain stimulation suppresses epileptic seizures in rats via inhibition of adenosine kinase and activation of adenosine A1 receptors

**DOI:** 10.1111/cns.14199

**Published:** 2023-04-05

**Authors:** Pandeng Xie, Siqi Liu, Qi Huang, Zhonghua Xiong, Jiahui Deng, Chongyang Tang, Ke Xu, Bo Zhang, Baijian He, Xiongfei Wang, Zhao Liu, Jing Wang, Jian Zhou, Yugang Guan, Guoming Luan, Tianfu Li, Feng Zhai

**Affiliations:** ^1^ Department of Neurosurgery, Center of Epilepsy, Beijing Institute for Brain Disorders, Beijing Key Laboratory of Epilepsy Research Sanbo Brain Hospital, Capital Medical University Beijing 100093 China; ^2^ Department of Brain Institute, Center of Epilepsy, Beijing Institute for Brain Disorders, Beijing Key Laboratory of Epilepsy Research Sanbo Brain Hospital, Capital Medical University Beijing 100093 China; ^3^ Department of Neurology, Center of Epilepsy, Beijing Institute for Brain Disorders Sanbo Brain Hospital, Capital Medical University Beijing 100093 China; ^4^ Department of Functional Neurosurgery, Neurological Center Beijing Children's Hospital, Capital Medical University, National Center for Children's Health Beijing 100045 China

**Keywords:** A1 receptors, adenosine kinase, deep brain stimulation, epilepsy

## Abstract

**Aims:**

Deep brain stimulation (DBS) of the anterior nucleus of the thalamus, is an effective therapy for patients with drug‐resistant epilepsy, yet, its mechanism of action remains elusive. Adenosine kinase (ADK), a key negative regulator of adenosine, is a potential modulator of epileptogenesis. DBS has been shown to increase adenosine levels, which may suppress seizures via A1 receptors (A_1_Rs). We investigated whether DBS could halt disease progression and the potential involvement of adenosine mechanisms.

**Methods:**

Control group, SE (status epilepticus) group, SE‐DBS group, and SE‐sham‐DBS group were included in this study. One week after a pilocarpine‐induced status epilepticus, rats in the SE‐DBS group were treated with DBS for 4 weeks. The rats were monitored by video‐EEG. ADK and A_1_Rs were tested with histochemistry and western blot, respectively.

**Results:**

Compared with the SE group and SE‐sham‐DBS group, DBS could reduce the frequency of spontaneous recurrent seizures (SRS) and the number of interictal epileptic discharges. The DPCPX, an A_1_R antagonist, reversed the effect of DBS on interictal epileptic discharges. In addition, DBS inhibited the overexpression of ADK and the downregulation of A_1_Rs.

**Conclusion:**

The findings indicate that DBS can reduce SRS in epileptic rats via inhibition of ADK and activation of A_1_Rs. A_1_Rs might be a potential target of DBS for the treatment of epilepsy.

## Graphical abstract

DBS therapy during epileptogenesis inhibits ADK overexpression and A_1_R downregulation in epileptic rats. DBS therapy might suppress seizures in epileptic rats at least partially via A_1_Rs.

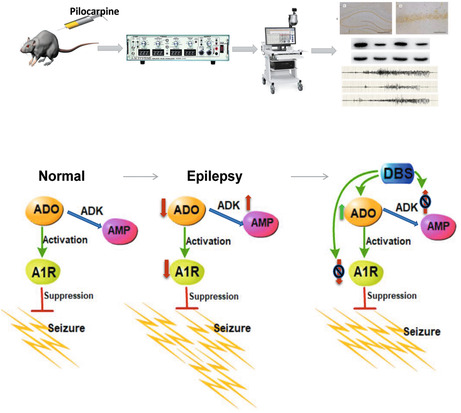

## 1 INTRODUCTION

Deep brain stimulation (DBS) is an effective neuromodulation therapy for patients with drug‐resistant epilepsy (DRE).^1^, ^2^, ^3^ The anterior nucleus of the thalamus (ANT) is one of the most common targets for DBS in the treatment of DRE, and its effectiveness has been proven.^4^, ^5^ ANT is a crucial node in the Papez circuit and has abundant projections to the frontal and temporal cortex.^6^, ^7^ This explains why ANT‐DBS most effectively reduces seizures originating in the temporal and frontal lobes.^8^

Pilocarpine injections in rodents provide a suitable model for studying DRE. Pilocarpine epileptic rat models have long‐term seizures and show extensive cell loss, which resembles what has been discovered in patients with temporal lobe epilepsy.^9^, ^10^, ^11^, ^12^ ANT‐DBS reduced the seizure rate and retarded the development of seizures in pilocarpine rats.^13^, ^14^, ^15^ However, the underlying mechanisms remain unclear.

Adenosine performs its anticonvulsant effects largely mediated via activation of adenosine A1 receptors (A_1_Rs),^16^, ^17^ whereas it acts as a pro‐epileptic signal through adenosine A2A receptors (A_2A_Rs).^18^, ^19^ The extracellular levels of adenosine in the brain are mainly curtailed by its major metabolic enzyme, adenosine kinase (ADK),^20^ which is mainly located in astrocytes in the adult brain.^21^ Overexpression of ADK in the epileptic hippocampus contributes to the development and progression of seizures.^22^, ^23^ Measured via microdialysis, DBS has been indicated to elevate adenosine levels in the hippocampus.^24^ In addition, ADK has been highly recognized as a hallmark of epileptogenesis.^22^, ^23^ We hypothesized that DBS might prevent epileptogenesis through the augmentation of adenosine signaling. In this study, a pilocarpine rat model was established and treated with ANT‐DBS to assess whether DBS can halt disease progression via potential underlying adenosine mechanisms.

## 2 METHODS

### 2.1 Animals

Adult Sprague–Dawley rats (male, 260‐300 g; Beijing Vital River Laboratory Animal Technology Co., Ltd.) were used for the experiments. Animals were housed individually in a controlled animal facility (22–24°C, 50%–60% humidity, and a 12‐h light/dark cycle) with freely accessed standard food and water. The study was approved by the Animal Experiments and Experimental Animal Welfare Committee of Capital Medical University (AEEI‐2021‐005). All animal experiments were carried out under the Beijing Guidelines for the Care and Use of Laboratory Animals.

### 2.2 Pilocarpine seizure induction and electrodes implantation

**FIGURE 1:**
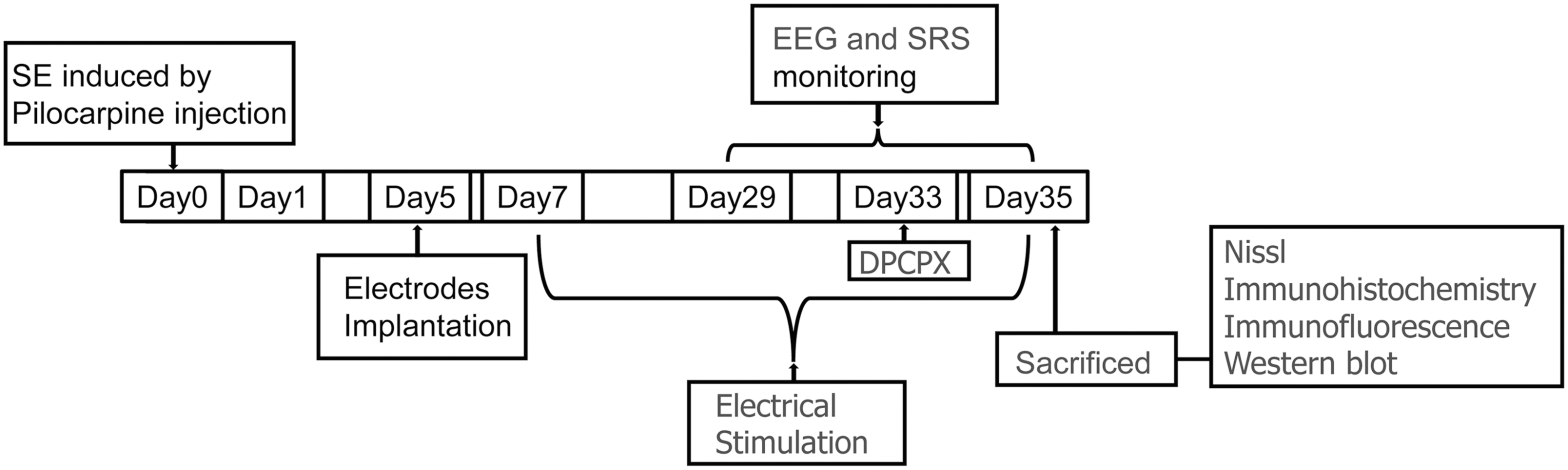
Experimental design of this study. SE, status epilepticus; SRS, spontaneous recurrent seizures.

Lithium chloride (3 mEq/kg; i.p.; Sigma‐Aldrich) and atropine (1 mg/kg, s.c.; Tianjin JinYao Pharmaceutical Industries Co., Ltd.) were injected 20 h and 30 min before pilocarpine (50 mg/kg, i.p.; Shanghai Aladdin Biochemical Technology Co., Ltd.), respectively. According to the Racine scale,^25^ rats that reached grade 4 or above and lasted for 1 h were considered to have acquired status epilepticus (SE). Rats with SE were treated with Diazepam (10 mg/kg, i.p.; Tianjin JinYao Pharmaceutical Industries Co., Ltd.) to halt seizures. The timeline for each operation is represented in Figure 1.

After 5 days, two bipolar tungsten electrodes (Cat No.796000, A‐M Systems) and a bipolar stainless‐steel electrode were respectively implanted into the bilateral ANT (AP: −1.5 mm, ML: ±1.5 mm, and DV: −5.2 mm relative to bregma) and the CA1 region of the right hippocampus (AP: −4.56 mm, ML: −2.7 mm, and DV: −3.0 mm relative to bregma) of rats, according to the Paxinos and Watson rat brain atlas.^26^

### 2.3 Electrical stimulation and video‐EEG recording

Electrical stimulation began 1 week after electrode implantation (parameters: 130 Hz, 90 μs, 100 μA; 8 h every day from 10:00 a.m. to 06:00 p.m.). Stimulation was delivered by pulse generators (S2100, A‐M Systems). Rats in the SE‐sham‐DBS group were connected to the unpowered pulse generator. Video‐electroencephalogram (Video‐EEG) data were collected and analyzed using NicoletOne EEG System (parameters: 1–70 Hz low‐ and high‐frequency filter, 15 mm/s recording speed; Natus). According to the Racine scale,^24^ SRSs (spontaneous recurrent seizures) on a scale of 3–5 and their duration were observed and recorded. Interictal epileptic discharges were calculated as reported in previous studies.^27^, ^28^ Animals in the SE‐DBS group were given an A_1_R antagonist, 8‐cyclopentyl‐1,3‐dipropyl xanthine (DPCPX, ab120396, 1 mg/kg, in 20% DMSO; Abcam) at second hour of electrical stimulation on day33. The video‐EEG was normally recorded during the process.

### 2.4 Tissue preparation

Half of the rats were perfused with 4% paraformaldehyde transcardially after being deeply anesthetized. The brains were submerged in 30% sucrose solution and then cut in a cryostat (thickness: 15 μm). After deep anesthetization, the remaining rats were killed to obtain hippocampi. All tissues were preserved at −80°C.

### 2.5 Histopathological assessment

**FIGURE 2:**
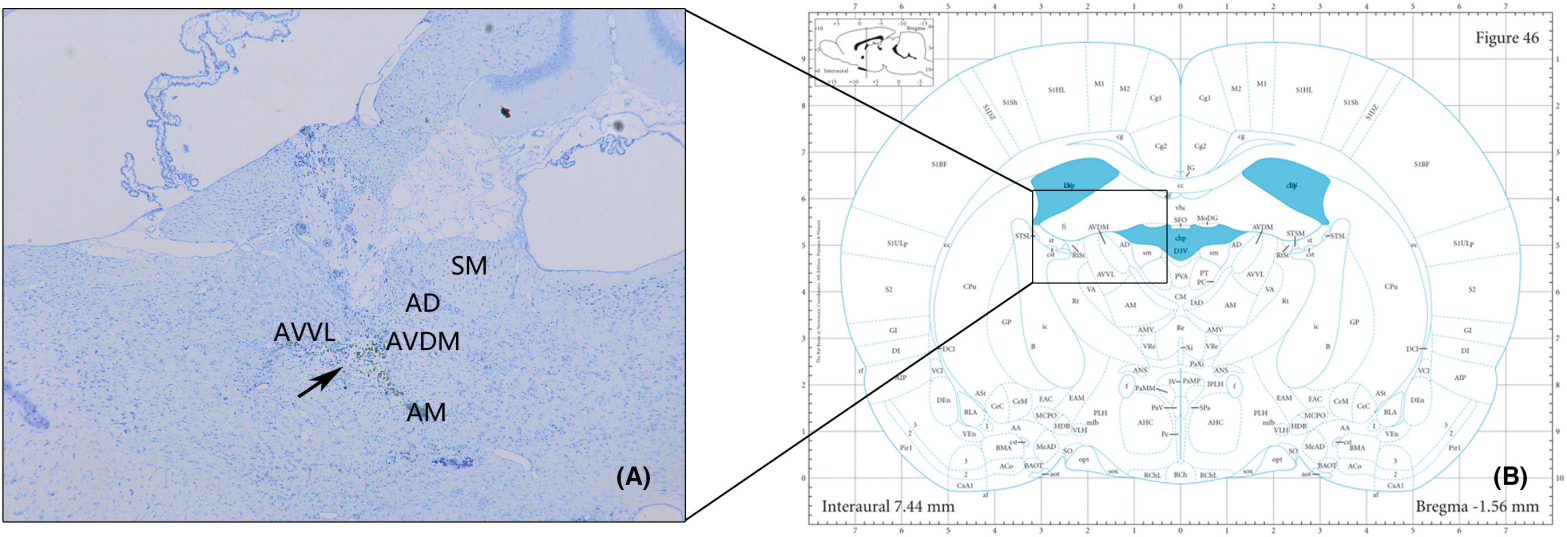
Location of the electrode tip. (A) Brain section with Nissl staining showing the location of the electrode tip (arrow). (B) A schematic representation of the coronal rat brain section showing the region in which the electrode tips were identified. AVVL, anteroventral thalamic nucleus, ventrolat; AVDM, anteroventral thalamic nucleus, dorsomed; AD, anterodorsal thalamic nucleus; AM, anteromedial thalamic nucleus; SM, stria medullaris.

Nissl staining was performed in sections containing electrode tracks to determine whether the electrodes were successfully implanted into the ANT (Figure 2). Nissl staining of sections containing the hippocampus was used to assess the condition of neurons.

Immunohistochemical staining was performed as described previously.^29^ After processing with 10% goat serum for 1 h at room temperature, samples were incubated with primary antibodies, including anti‐A_1_R (monoclonal rabbit ab124780,1:100; Abcam) and anti‐ADK (polyclonal rabbit, 1:200; provided by Professor Detlev Boison, Department of Neurosurgery, Robert Wood Johnson & New Jersey Medical Schools, Rutgers University).^29^, ^30^, ^31^, ^32^ Subsequently, the sections were exposed to the secondary antibody (goat, PV‐9000, 100 μL per section; ZSGB‐BIO) for 1 h, and 3,3‐diaminobenzidine (DAB; 1–3 min) was used as the chromogen. A light microscope (Leica DM 3000LED, Leica Microsystems), was used to count the cells.

Double‐label immunofluorescence was performed as described previously.^29^, ^32^ After incubation with anti‐GFAP (monoclonal mouse, 1:1000; ab10062, Abcam) and anti‐ADK (polyclonal rabbit, 1:200; provided by Professor Detlev Boison), sections were incubated with Alexa Fluor 594 and Alexa Fluor 488 (anti‐rabbit IgG or anti‐mouse IgG; Thermo Fisher Scientific) for 1 h, and nuclei were stained with 4′,6‐diamidino‐2‐phenylindole (DAPI). Fluorescence microscopy (Olympus BX51; Olympus) was used for image acquisition.

### 2.6 Western blot

The experimental process of western blot was carried out as described previously^32^ with modification. The samples of the hippocampus were homogenized in a lysis buffer solution. Sodium dodecyl sulfate (SDS) polyacrylamide gel was used to separate proteins (30 μg/lane). Subsequently, proteins were transferred to a polyvinylidene fluoride (PVDF) membrane (0.22 μm, Millipore Corp). After soaking in 8% milk for 90 min, membranes were incubated with anti‐ADK (polyclonal rabbit, 1:200; from Professor Detlev Boison, Department of Neurosurgery, Robert Wood Johnson & New Jersey Medical Schools, Rutgers University) or anti‐A_1_R (monoclonal rabbit ab124780, 1:100, Abcam).

Anti‐β‐actin (monoclonal mouse, 1:1000; Cell Signaling Technology) was used to normalize results. After incubating with HRP‐conjugated secondary antibodies (1:5000; Applygen Technologies Inc.), proteins were visualized using enhanced chemiluminescence reagents (Applygen, Technologies Inc.). Autoradiography films were scanned with a luminescent image analyzer (LAS‐3000; Fujifilm). The quantitative analysis process of protein strips was performed using ImageJ software.

### 2.7 Statistics analyses

Statistical analysis was performed by GraphPad Prism 8.4.2 software (GraphPad Software). Results are displayed as mean ± SEM. All data were subject to tests for normality using the Shapiro–Wilk test and all *p*‐values were > 0.05. One‐way analysis of variance (ANOVA) followed by Tukey's multiple comparisons test was used for multiple groups. *p* < 0.05 was considered statistically significant.

## 3 RESULTS

### 3.1 DBS reduces the frequency of SRS in epileptic rats

**FIGURE 3:**
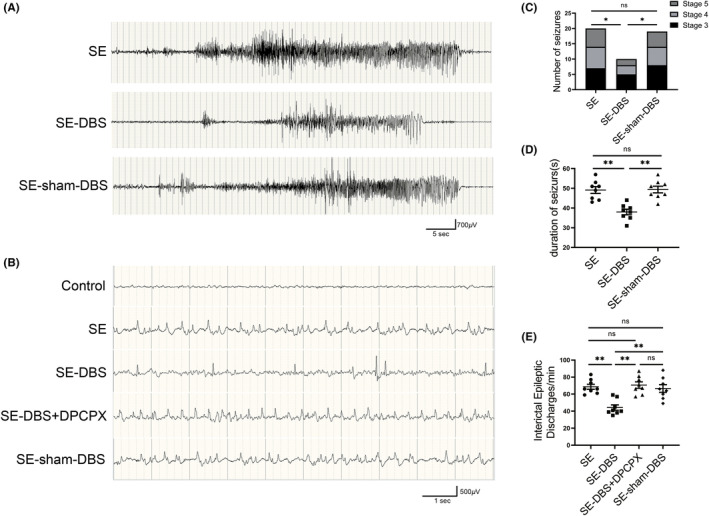
Effectiveness of DBS on the SRS and epileptic discharge by EEG. (A) Representative EEG recordings from the hippocampus showed spontaneous electrographic seizures in the SE, SE‐DBS, and SE‐sham‐DBS groups. (B) Representative EEG recordings from the hippocampus showed interictal epileptic discharges in the SE, SE‐DBS, and SE‐sham‐DBS groups; interictal epileptic discharges of animals injected with DPCPX in SE‐DBS group are shown in column SE‐DBS + DPCPX. (C) The number of SRS recorded in the last week (Day 29–35) of the DBS period, only the seizures of stage 3 or greater were counted according to the Racine scale, demonstrating that the rats of the SE‐DBS group had a lesser number of seizures than those of the SE‐sham‐DBS group (*p < 0.05, *n* = 8). (D) The duration of SRS was compared between each group, demonstrating that the mean SRS duration of the SE‐DBS group was significantly shorter than that of the SE‐sham‐DBS group (**p < 0.01, *n* = 8). (E) Quantification analysis showed that the number of interictal epileptic discharges in the SE‐DBS group was significantly lower than that in the SE‐sham‐DBS group (**p < 0.01, *n* = 8). A total duration of 30 min after the DPCPX injection, the number of interictal epileptic discharges in the SE‐DBS group increased significantly (**p < 0.01, *n* = 8).

A total of 63 rats were included in this study. Fifty‐five of these animals were injected with pilocarpine solution. Forty‐four rats achieved SE with grades 4–5 on the Racine scale, 17 died of SE or malnutrition, and three were excluded for the position of electrode implantation missing the ANT. In the final experiment, eight animals were included in each group. To evaluate the effect of DBS on the development of epilepsy in rats, we recorded episodes and video‐EEG recordings in the fifth week after SE. No seizures or interictal epileptic discharge were seen in the rats in the control group during the experimental period. The mean number of SRSs in the SE‐DBS group was reduced by 47.37% compared to the SE‐sham‐DBS group (Figure 3C, 1.25 ± 0.25 and 2.38 ± 0.32 times/day, respectively, *p* < 0.05). No significant differences were observed between the SE and SE‐sham‐DBS groups. The mean seizure duration of the SE‐DBS group was significantly shorter than that of the SE‐sham‐DBS group (Figure 3D, 38.00 ± 1.41 and 49.38 ± 1.68, respectively, *p* < 0.01). No significant difference was observed between the SE group and the SE‐sham‐DBS group. Our results demonstrated that DBS treatment significantly reduced the number of SRSs in epileptic rats after SE and shortened the duration of seizures.

### 3.2 DBS reduces the frequency of interictal epileptic discharge in epileptic rats, and DPCPX can block the antiepileptic effect of DBS

Interictal epileptic discharges were calculated to evaluate the therapeutic effect of DBS in epileptic rats. Interictal epileptic discharges in the EEG are shown in Figure 3B. The number of interictal epileptic discharges in the SE‐DBS group was significantly lower than that in the SE‐sham‐DBS group (Figure 3E, 44.25 ± 3.17 and 66.63 ± 4.50 times/min, respectively, *p* < 0.01). No significant difference was found between the SE and SE‐sham‐DBS groups. A total of 30 min after the DPCPX injection, the number of interictal epileptic discharges in the SE‐DBS group increased significantly (Figure 3E, 70.63 ± 3.71, *p* < 0.01). This suggests that DPCPX may transiently block the antiepileptic effect of DBS.

### 3.3 DBS decreases the loss of hippocampal neurons in epileptic rats

**FIGURE 4:**
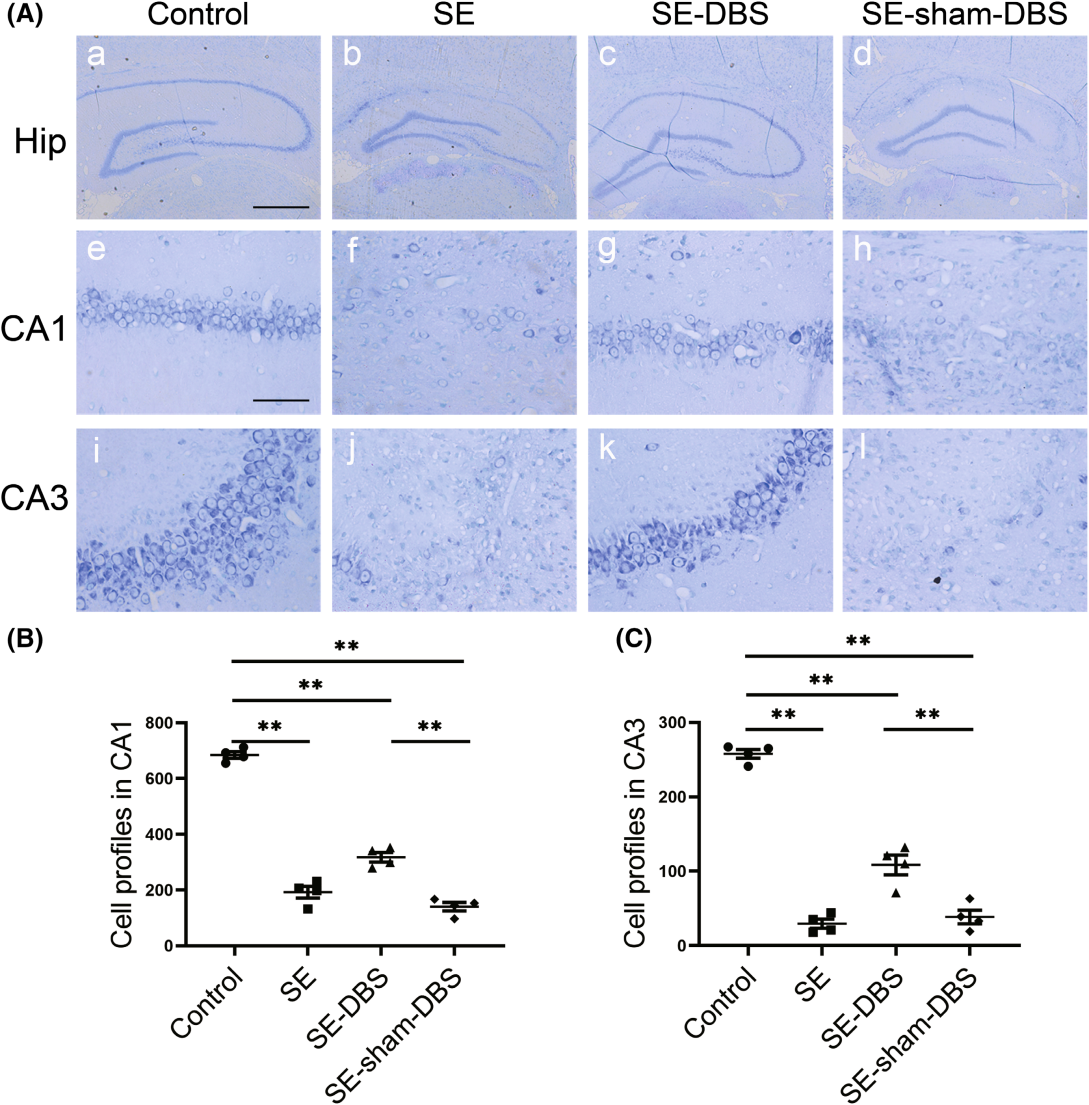
Effect of DBS on neuronal loss in the hippocampus. Nissl‐stained sections through the hippocampus were used to assess pilocarpine‐induced neuronal loss at 5 weeks (Day 29–35) after SE. (A) No apparent neuronal loss was discovered in the CA1 and CA3 regions in control group (a, e, and i, respectively); Obvious neuronal loss within CA1 and CA3 regions were observed in SE (b, f, and j, respectively), SE‐DBS (c, g, and k, respectively), and SE‐sham‐DBS (d, h, and l, respectively) groups, compared with control group. (B and C) Compared to the SE‐sham‐DBS group, significantly less neuronal loss was observed within hippocampal CA1 and CA3 regions in the SE‐DBS group (**p < 0.01, *n* = 4). Scale bars = 500 μm (a–d) and 50 μm (e–l).

To assess the protective effect of DBS on hippocampal neuronal loss in pilocarpine rats during epileptogenesis, we counted the number of hippocampal neurons across each group. We found that there was no apparent neuronal loss in the CA1 and CA3 regions in the control group (Figure 4A,a,e,i). Compared with control group, neuronal loss within CA1 and CA3 regions was remarkably observed in SE (Figure 4A,b,f,j), SE‐DBS (Figure 4A,c,g,k), and SE‐sham‐DBS (Figure 4A,d,h,l) groups. Compared to the SE‐sham‐DBS group, significantly less neuronal loss was observed within hippocampal CA1 and CA3 regions in the SE‐DBS group (Figure 4B,C, *p* < 0.01). No significant difference in the neuronal loss within CA1 and CA3 regions of the hippocampus was revealed between SE and SE‐sham‐DBS groups (Figure 4B,C, *p* > 0.05). The data showed that DBS can prevent the loss of hippocampal neurons in epileptic rats.

### 3.4 DBS inhibits the overexpression of ADK

**FIGURE 5:**
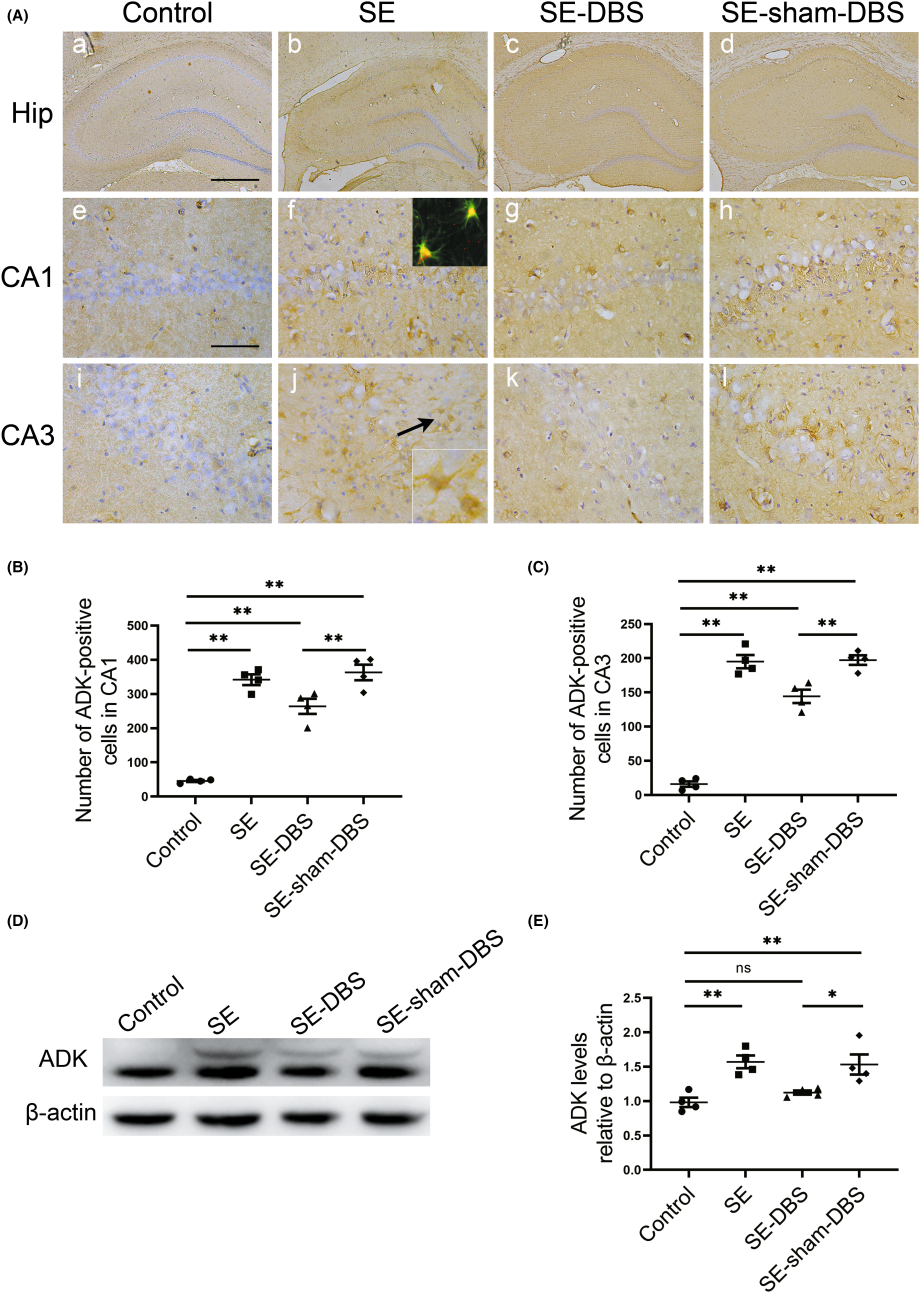
Effect of DBS on ADK levels in the hippocampus. Immunohistochemical staining of ADK, double‐label immunofluorescence staining of ADK and GFAP, and analysis of ADK by western blotting were evaluated in the hippocampus at 5 weeks after SE. (A) ADK immunoreactivity was present in sparse astroglial cells with only a weak staining and lack of ADK immunoreactivity in neuronal cells in control group (a, e, and i, respectively); Compared with control group, overexpression of ADK was shown in the reactive astroglial cells within CA1 and CA3 regions of the hippocampus in SE group (b, f, and j, respectively), SE‐DBS group (c, g, and k, respectively), and SE‐sham‐DBS group (d, h, and l, respectively); (f) Overlay of GFAP (green) and ADK (red) immunofluorescence(inset); (j) ADK‐positive cells (arrow, inset). (B and C) The number of ADK‐positive cells within CA1 and CA3 regions of the hippocampus in the SE‐DBS group was fewer than that in the SE‐sham‐DBS group (**p < 0.01, *n* = 4). (D) Analysis of ADK in the hippocampus by western blot (original, uncropped image see Figure S1). (E) In SE and SE‐sham‐DBS groups, ADK levels in the hippocampus were elevated compared with the control group (**p < 0.01, *n* = 4); In SE‐DBS group, less ADK were observed compared with the SE‐sham‐DBS group (*p < 0.05, *n* = 4). Scale bars = 500 μm (a–d) and 50 μm (e–l).

Similar to previous studies,^30^, ^32^ in control group, ADK immunoreactivity was present in sparse astroglial cells with only weak staining (Figure 5A,a,e,i) and a lack of ADK immunoreactivity in neuronal cells. Compared with control group, overexpression of ADK was shown in the reactive astroglial cells within CA1 and CA3 regions of the hippocampus in SE (Figure 5A,b,f,j, inset arrow), SE‐DBS (Figure 5A,c,g,k), and SE‐sham‐DBS (Figure 5A,d,h,l) groups. The number of ADK‐positive cells within the CA1 and CA3 regions of the hippocampus in the SE‐DBS group was lower than that in the SE‐sham‐DBS group (Figure 5B,C, *p* < 0.01). No statistical difference in the number of ADK‐positive cells within hippocampal CA1 and CA3 regions was discovered across the SE and SE‐sham‐DBS groups (Figure 5B,C, *p* > 0.05).

Western blot analysis was performed to quantify the amount of ADK in total homogenates of the hippocampus from rats in each group (Figure 5D). Compared with control group, there were significantly greater ADK levels in the SE and SE‐sham‐DBS groups (Figure 5E, *p* < 0.01). The levels of ADK in the SE‐DBS group were considerably lower than that in the SE‐sham‐DBS group (Figure 5E, *p* < 0.05). No significant difference in ADK levels was detected between SE and SE‐sham‐DBS groups (Figure 5E, *p* > 0.05). Quantitative analysis of ADK confirmed that DBS treatment can significantly inhibit the increased density of ADK in the hippocampus of epileptic rats.

### 3.5 DBS attenuates the downregulation of A_1_Rs

**FIGURE 6:**
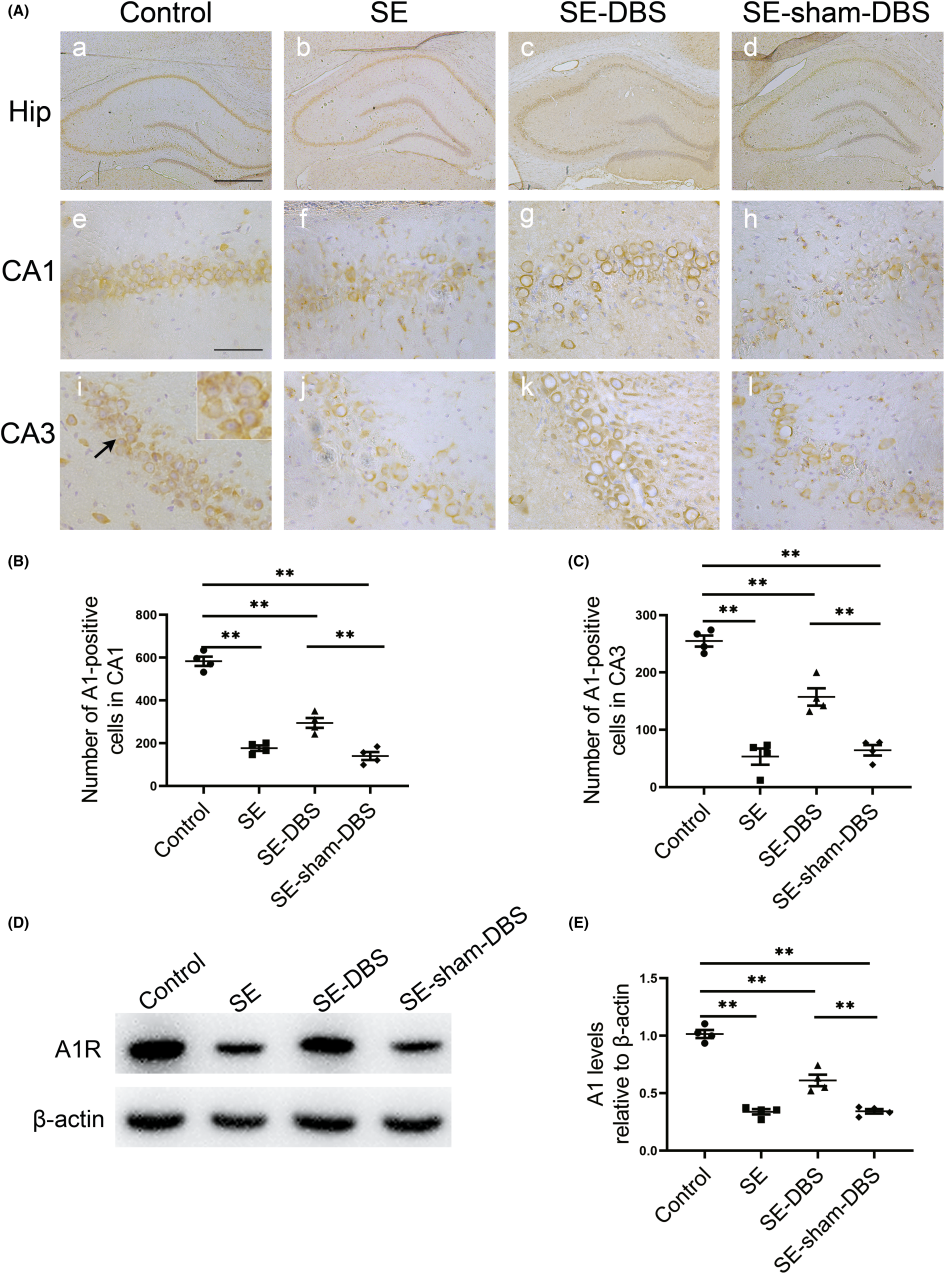
Effect of DBS on A_1_R levels in the hippocampus. Immunohistochemical staining of A_1_Rs and analysis of A_1_Rs by western blotting were evaluated in the hippocampus at 5 weeks after SE. (A) In control group, A_1_R immunoreactivity was present exclusively in neuronal cells in the hippocampus (a, e, and i, respectively, arrow, insect); Compared with the control group, the levels of A_1_Rs were significantly downregulated within CA1 and CA3 regions of the hippocampus in SE group (b, f, and j), SE‐DBS group (c, g, and k, respectively) and SE‐sham‐DBS group (d, h, and l, respectively). (B and C) Quantitative analysis revealed a significant increase in the number of A_1_R‐positive cells within CA1 and CA3 regions of the hippocampus in the SE‐DBS group, compared with the SE‐sham‐DBS group (**p < 0.01, *n* = 4). (D) Analysis of A_1_Rs in the hippocampus by western blot (original, uncropped image see Figure S2). (E) Compared with control group, there were significantly lower levels of A_1_Rs in SE, SE‐DBS, and SE‐sham‐DBS groups (**p < 0.01, *n* = 4); The levels of A_1_Rs within the hippocampus in the SE‐DBS group were significantly higher than that in the SE‐sham‐DBS group (**p < 0.01, *n* = 4). Scale bars = 500 μm (a–d) and 50 μm (e–l).

The immunostaining of A_1_Rs was studied by immunohistochemistry in brain specimens of rats in each group. In control group, A_1_R immunoreactivity was present exclusively in neuronal cells in the hippocampus (Figure 6A,a,e,i, insect arrow). Compared with the control group, the levels of A_1_Rs were significantly downregulated within CA1 and CA3 regions of the hippocampus in SE (Figure 6A,b,f,j), SE‐DBS (Figure 6A,c,g,k), and SE‐sham‐DBS (Figure 6A,d,h,l) groups. Quantitative analysis revealed a significant increase in the number of A_1_R‐positive cells within CA1 and CA3 regions of the hippocampus in the SE‐DBS group, compared with the SE‐sham‐DBS group (Figure 6B,C, *p* < 0.01). No statistical difference was found between SE and SE‐sham‐DBS groups (Figure 6B,C, *p* > 0.05).

Western blot analysis was also performed to quantify the amount of A_1_Rs in total homogenates of the hippocampus in each group (Figure 6D). Compared with control group, there was a significantly lower level of A_1_Rs in SE, SE‐DBS, and SE‐sham‐DBS groups (Figure 6E, *p* < 0.01). The levels of A_1_Rs in the SE‐DBS group were significantly higher than that in the SE‐sham‐DBS group (Figure 6E, *p* < 0.01). No significant difference was detected between SE and SE‐sham‐DBS groups (Figure 6E, *p* > 0.05). These results suggest that DBS therapy during epileptogenesis can attenuate the downregulation of A_1_Rs in epileptic rats.

## 4 DISCUSSION

### 4.1 DBS therapy delays disease development during epileptogenesis

In recent decades, research on electrical stimulation of specific regions of the brain has been conducted.^33^ There is accumulating evidence that ANT‐DBS has therapeutic effects on DRE.^1^, ^3^, ^13^ In this study, we adopted the pilocarpine epilepsy rat model with similar neuropathological and electrophysiological characteristics of DRE patients. Epileptogenesis is a process in which a healthy brain becomes an epileptic brain.^34^ Via video‐EEG monitoring, we found that high‐frequency ANT‐DBS therapy during epileptogenesis can not only reduce the frequency of SRS, but also shorten the duration of seizures (decrease the severity of seizures) (Figure 3). In addition, the number of interictal epileptic discharges of the epileptic rats was significantly reduced as well after DBS treatment (Figure 3), which is in accordance with the previous research.^35^, ^36^ DBS delays epileptogenesis by interfering with the propagation of epileptiform activity when applied during the interictal period and alleviates seizures by delaying the modification of microcircuit recruitment.^37^ Epileptogenesis can result in remarkable neuronal loss in the hippocampus through an array of complex pathological alterations.^38^, ^39^, ^40^ DBS can reduce neuronal loss in the hippocampus of epileptic rats.^41^, ^42^ The inhibition of apoptosis in the hippocampus mediates the neuroprotective effect.^41^ In this study, DBS therapy initiated in epileptogenesis can provide remarkable neuroprotection (Figure 4).

### 4.2 DBS therapy increases the adenosine signaling via inhibition of ADK

Adenosine is mainly removed by ADK,^20^ which is principally expressed in astrocytes in the adult brain.^21^ Astrocyte‐expressed ADK, a key negative regulator of the brain inhibitory molecule adenosine, is a potential predictor and modulator of epileptogenesis.^29^ During epileptogenesis, ADK overexpression in the hippocampus promotes the development and progression of seizures.^22^, ^23^ Upregulation of ADK has been highly regarded as a common pathologic hallmark of epilepsy.^23^, ^29^, ^43^, ^44^ In addition, DBS can promote ATP release and induce adenosine accumulation in the brain.^24^, ^45^, ^46^, ^47^ In this study, DBS therapy exerts remarkable inhibition of ADK upregulation in epileptic rats (Figure 5). Given that ADK is the main scavenging enzyme of adenosine, inhibiting its overexpression interferes with adenosine elimination, which is a crucial factor promoting adenosine accumulation. It has been demonstrated that therapeutic adenosine augmentation can prevent epileptogenesis.^48^ The long‐term accumulation of adenosine may inhibit epilepsy progression in epileptic rats.

In addition, ADK is involved in epileptogenesis by regulating the DNA methylation pathway.^49^ DNA methylation in the central nervous system participates in epilepsy formation by regulating neuronal networks and synaptic plasticity.^48^, ^50^, ^51^ Locally increasing the level of adenosine can reduce or reverse abnormal methylation of DNA and inhibit the development of epilepsy. Thus, the decrease in ADK levels or an increase in adenosine in the brain reverses the equilibrium of the methylation pathway, which provides therapeutic value as regards the treatment of epileptogenesis.^48^ Therefore, we hypothesized that DBS therapy might retard epileptogenesis via the inhibition of ADK. It is worth noting that the efficacy of DBS gradually increases with therapeutic time and is far more remarkable in the long term.^3^ DBS provides long‐term adenosine enhancement in the brain, which might explain the characteristic of long‐term DBS efficacy.

### 4.3 DBS therapy inhibits seizures via A_1_Rs

The actions of adenosine in the brain are mainly mediated by A_1_Rs and A_2A_Rs, while the A_2B_Rs and A_3_Rs are still poorly studied.^52^ As an endogenous substance, adenosine acts as an anticonvulsant mostly through A_1_Rs.^16^, ^17^ Activation of the presynaptic A_1_Rs reduces the release of excitatory neurotransmitters, especially glutamate,^53^ while the postsynaptic A_1_Rs hyperpolarize neurons through G protein‐coupled potassium channels, which inhibits the excitability of the hippocampus.^54^ In this study, downregulation of hippocampal A_1_Rs was observed in epileptic rats. DBS therapy attenuates the downregulation of hippocampal A_1_Rs (Figure 6). It is worth mentioning that the staining shows a predominant A_1_R staining in the cell bodies (Figure 6), while A_1_Rs are very well established to be mainly synaptically located.^55^ It is probably because antibodies in our study do not reach synaptic epitopes in the process of immunohistochemical staining.

In a rat kindling model, the reduction of A_1_R levels and the loss of endogenous adenosine‐based seizure control indicated a failure of endogenous adenosine‐mediated seizure control mechanisms.^56^ A_1_R knockout mice developed spontaneous electrographic seizures and lethal SE following injection with kainic hippocampal acid in the hippocampus or traumatic brain injury.^57^ In pilocarpine‐induced epileptic rats, the levels of A_1_Rs in the hippocampus showed a significant decrease in epileptogenesis.^58^ Actually, in several other previous studies, A_1_Rs density in the hippocampus is decreased in kainic acid‐induced and kindling models.^56^, ^59^, ^60^ These studies suggest that the pathophysiology of epilepsy is associated with abnormal A_1_R signaling.

Stimulation‐induced adenosine release in ferret slices was able to abolish spontaneous spindle oscillations.^46^ In pilocarpine‐induced epileptic rats, DBS therapy increased adenosine levels in the hippocampus.^24^ In vitro study showed that the excitability reduction of DBS therapy in rats pretreated with A_1_R antagonists was completely eliminated and was strongly enhanced by A_1_R agonists.^24^ To confirm the effect of A_1_Rs on the therapeutic effect of DBS therapy in vivo, the rats in the SE‐DBS group were administrated with an A_1_R antagonist DPCPX in this study. The frequency of interictal epileptic discharges in the SE‐DBS group was increased and restored to the level of SE‐group following injection of DPCPX (Figure 3B,E), thereby revealing that the inhibition of interictal epileptic discharges in the hippocampus of epileptic rats by DBS treatment was completely eliminated by the loss of A_1_R signal. Therefore, DBS therapy might suppress seizures in epileptic rats at least partially via A_1_Rs.

## 5 CONCLUSION

Our data reveal that DBS therapy initiating in epileptogenesis can dampen disease development, inhibit hippocampal neuron loss, and inhibit ADK overexpression and A_1_R downregulation in epileptic rats. A_1_Rs may be potential targets of DBS for the treatment of epilepsy. The effectiveness of DBS therapy initiating in chronic stage of epilepsy needs further study in the future.

## CONFLICT OF INTEREST STATEMENT

The authors have nothing to disclose and declare no conflicts of interest.

## Supporting information


Data S1:

## Data Availability

The data that support the findings of this study are available from the corresponding author upon reasonable request.
