# Supplementary material for: Deep brain stimulation suppresses epileptic seizures in rats via inhibition of adenosine kinase and activation of adenosine A1 receptors
Source: CNS Neurosci Ther. 2023 Apr 5;29(9):2597–607. doi: 10.1111/cns.14199 (PMC10401158; doi:10.1111/cns.14199)
Supplement: Supplementary file 1 — Data S1: [file CNS-29-2597-s001.pdf]

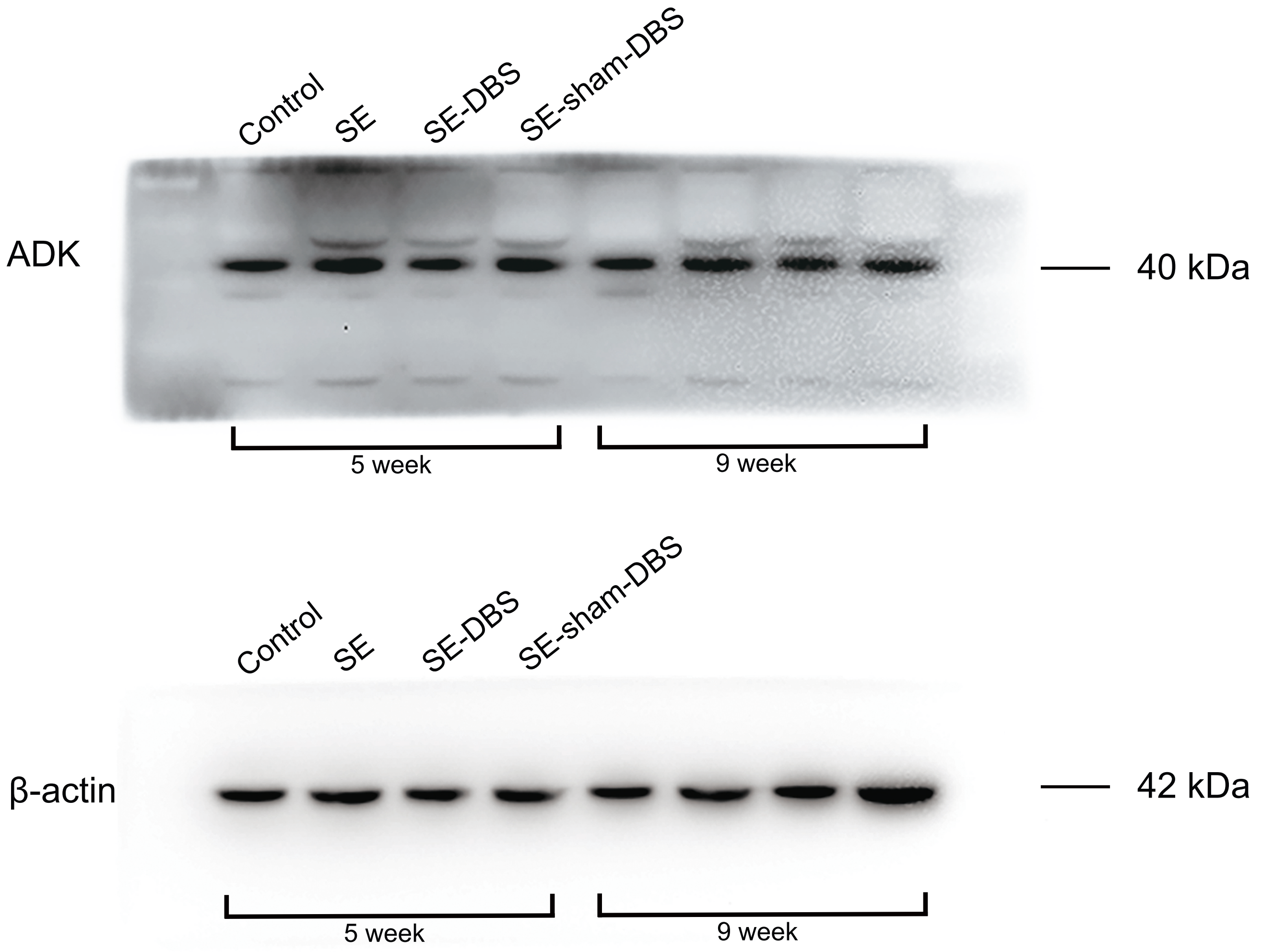

Fig. S1. Full unedited gel/blot for the analysis of ADK in the hippocampus in western blot.

The “5 week” sections of ADK and β-actin blots were cropped and analysied in Fig. 5D in the manuscript to show the levels of ADK in the hippocampus of epileptic rats.

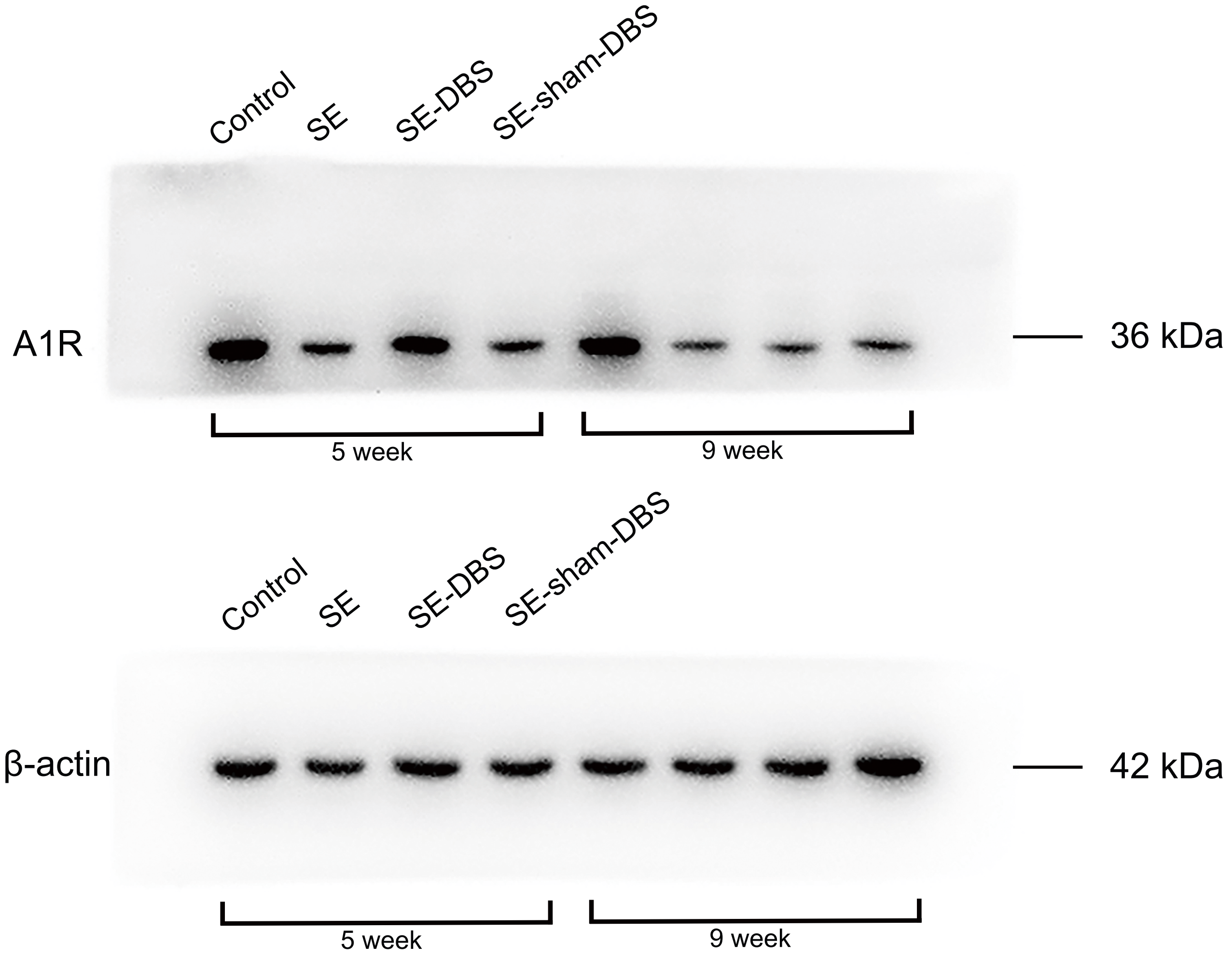

Fig. S2. Full unedited gel/blot for the analysis of A1R in the hippocampus in western blot.

The “5 week” sections of A1R and β-actin blots were cropped and analysied in Fig. 6D in the manuscript to show the levels of A1R in the hippocampus of epileptic rats.
